# Exact Solutions for the Non-Isothermal Poiseuille Flow of a FENE-P Fluid

**DOI:** 10.3390/polym17172343

**Published:** 2025-08-29

**Authors:** Evgenii S. Baranovskii

**Affiliations:** Department of Applied Mathematics, Informatics and Mechanics, Voronezh State University, 394018 Voronezh, Russia; esbaranovskii@gmail.com

**Keywords:** polymer fluids, micro–macro models, FENE-P model, Maxwell model, exact solutions, plane Poiseuille flow, heat and mass transfer, viscous dissipation, Navier slip, threshold slip condition

## Abstract

In the present article, we study a nonlinear mathematical model for the steady-state non-isothermal flow of a dilute solution of flexible polymer chains between two infinite horizontal plates. Both plates are assumed to be at rest and impermeable, while the flow is driven by a constant pressure gradient. The fluid rheology model used is FENE-P type. The flow energy dissipation (mechanical-to-thermal energy conversion) is taken into account by using the Rayleigh function in the heat transfer equation. On the channel walls, we use one-parameter Navier’s conditions, which include a wide class of flow regimes at solid boundaries: from no-slip to perfect slip. Moreover, we consider the case of threshold-type slip boundary conditions, which state the slipping occurs only when the magnitude of the shear stresses overcomes a certain threshold value. Closed-form exact solutions to the corresponding boundary value problems are obtained. These solutions represent explicit formulas for the calculation of the velocity field, the temperature distribution, the pressure, the extra stresses, and the configuration tensor. The results of the work favor better understanding and more accurate description of complex dynamics and energy transfer processes in FENE-P fluid flows.

## 1. Introduction

It is well known that many real fluids and fluid-like materials do not satisfy Newton’s law of viscosity, which corresponds to a linear relation between the shear stress and rate-of-strain tensors. For example, media containing high molecular weight polymers such as polymer solutions and melts, or of multiphase media like emulsions and slurries often exhibit complex non-Newtonian characteristics like memory effects, viscoelasticity, variable viscosity, etc. Such fluids obey complex (usually, nonlinear) constitutive equations and are called *non-Newtonian*.

Heat and mass transfer problems involving non-Newtonian fluid flows in channels/pipes and network systems have attracted growing interest in the mathematical theory and engineering sciences. This is due to the fact that many biological fluids (such as blood, mucus, etc.) and fluids used in engineering, the food industry, or agriculture fall into the class of non-Newtonian fluids.

Since the departure from the “Newtonian” behavior occurs in various ways, numerous different models have been proposed to describe flows of non-Newtonian fluids. Let us mention here, for example, differential and integral models as well as micro–macro models, which are based on the kinetic formulation of the probability distribution function (see [1,2,3] for details).

In this paper, we deal with the FENE-P model for dilute solutions of flexible polymer chains. This model was suggested by Peterlin [4] as a macroscopic approximation of the finitely extensible nonlinear elastic (“FENE”) dumbbell model, which is one of the most used micro–macro models in the theory of polymer dynamics [1,5,6]. Recall that in the framework of the FENE model, a polymer is idealized as an elastic dumbbell that consists of two beads joined by a spring (see [7] for some physical introduction to the model).

The FENE-P model is well appropriate for describing the dynamics of dilute polymer solutions due to its ability to take into account nonlinear effects that arise from the finite extensibility of the polymer chains [8]. In particular, this is related to Newtonian solvents including some (small) amounts of dissolved polymer. Flows of such media exhibit dramatically diverse properties from the ones for the corresponding pure Newtonian liquid flows in view of the creation of sustained filament-type structures under stretching.

The mathematical formulation of the non-isothermal incompressible FENE-P system reads as follows:

(1) $$\begin{array}{c} \rho \left(\frac{\partial \vec{v}}{\partial t}+\mathrm{div}\left(\vec{v}⊗\vec{v}\right)\right)=\mathrm{div}\;T+\rho \vec{g}, \end{array}$$

(2) $$\begin{array}{c} \nabla \cdot \vec{v}=0, \end{array}$$

(3) $$\begin{array}{c} T=-pI+S, \end{array}$$

(4) $$\begin{array}{c} S=\frac{\eta }{\lambda }\left(\frac{A}{1-\frac{\mathrm{tr}(A)}{L^{2}}}-aI\right), \end{array}$$

(5) $$\begin{array}{c} \overset{\nabla }{A}=-\frac{1}{\lambda }\left(\frac{A}{1-\frac{\mathrm{tr}(A)}{L^{2}}}-aI\right), \end{array}$$

(6) $$\begin{array}{c} \rho \left(\frac{\partial \theta }{\partial t}+\left(\vec{v}\cdot \nabla \right)\theta \right)-\kappa \nabla^{2}\theta =\psi +\Phi \left(T,D\left(\vec{v}\right)\right), \end{array}$$

where

- ρ is the fluid density, ρ>0;
- $\vec{v}$ is the velocity vector;
- T is the Cauchy stress tensor;
- *p* is the pressure;
- I is the identity tensor;
- S is the extra stress tensor;
- $\vec{g}$ is the external force applied to the fluid;
- the symbol ⊗ denotes the tensor product of vectors, $\vec{v}⊗\vec{w}\overset{\mathrm{def}}{=}{\left(v_{i}w_{j}\right)}_{i,j=1}^{3}$ for $\vec{v},\vec{w}\in R^{3}$;
- A is the configuration tensor, which is positive-definite;
- tr(A) denotes the trace of A;
- η is the polymer viscosity, η>0;
- λ is the relaxation time, λ>0;
- *L* denotes a dimensionless parameter ($L>\sqrt{3}$) that characterizes the extensibility of polymer chains;
- $a\overset{\mathrm{def}}{=}\frac{1}{1-\frac{3}{L^{2}}}$ is an additional parameter, which is not independent;
- the differential operators ∇, $\nabla^{2}$ and “div” are the gradient, the Laplacian and the divergence, respectively, with respect to the space variables *x*, *y*, *z*;
- $\overset{\nabla }{A}$ stands for frame-invariant Oldroyd’s upper convected derivative of A,
  (7) $$\overset{\nabla }{A}\overset{\mathrm{def}}{=}\frac{\partial A}{\partial t}+\left(\vec{v}\cdot \nabla \right)A-\left(\nabla \vec{v}\right)A-A{\left(\nabla \vec{v}\right)}^{⊤};$$
- θ is the temperature;
- κ is the thermal conductivity, κ>0;
- ψ is the heat source intensity;
- Φ is the Rayleigh function describing the effect of viscous dissipation (mechanical-to-thermal energy conversion), $\Phi \left(T,D\left(\vec{v}\right)\right)\overset{\mathrm{def}}{=}\frac{1}{c_{p}}T:D\left(\vec{v}\right);$
- $c_{p}$ is the heat capacity of the fluid, $c_{p}>0$;
- $D(\vec{v})$ is the deformation rate tensor, $D\left(\vec{v}\right)\overset{\mathrm{def}}{=}\frac{1}{2}\left(\nabla \vec{v}+{\left(\nabla \vec{v}\right)}^{⊤}\right)$;
- the colon symbol: denotes the scalar product of tensors.

In the literature, sometimes it is assumed that the value of the parameter *L* is sufficiently large, which implies the equality a=1. However, following [9,10], we will not accept this assumption in the present paper. Also notice that the FENE-P model is considered without taking into account the solvent viscosity and the material properties like the polymer viscosity and the relaxation time are assumed to be independent of temperature.

Equation (1) is the general equation of motion for a continuous medium in the Cauchy form. Relation (2) represents the incompressibility condition. Coupled relations (3)–(5) describe fluid rheology, that is, an implicit differential constitutive model is considered. Using Oldroyd’s convective derivative in (5) allows the flow model to be consistent with the fundamental principle of material frame indifference (material objectivity); see [11] for details. Equation (6) represents the energy balance law, taking into account the viscous dissipation effect by the Rayleigh function Φ.

The investigation of mathematical problems related to the FENE model and its approximations is rather difficult even under simplifying assumptions. Nowadays, there are many open and challenging problems in this direction. Some results on the well-posedness for the corresponding governing equations are given in the papers [12,13,14,15,16,17,18,19,20]. Stability issues and large-time behavior for the 2D FENE model near an equilibrium are discussed in the work [21]. We refer interested readers to Le Bris & Lelièvre [1] as well as Li & Zhang [22] for detailed mathematical overviews on various micro–macro models of complex fluids. Also note that most of the known results are obtained for the isothermal case.

The analysis of dynamics of viscoelastic media with nonlinear constitutive equations is of fundamental interest for the scientific community because of their essential complexity as a direct consequence of non-Newtonian rheology. In studying such models, exact (analytical and semi-analytical) solutions play a special, irreplaceable role. They promote a deeper understanding of various qualitative features of both steady-state and time-dependent flows (for example, stability-instability, bifurcations, blow-up regimes, etc.) and make it possible to better estimate the applicability area of flow models. Moreover, as is known, exact solutions are very useful for testing numerical, asymptotic, and approximate analytical methods. Note that even solutions that do not have a clear physical interpretation can be applied for testing.

The main aim of the present paper is to obtain exact solutions for the non-isothermal steady-state Poiseuille flow of a FENE-P fluid confined between two parallel plates.

We use various types of boundary conditions, not limited to the standard no-slip condition. The importance of taking into account slip effects in modeling of fluid dynamics and heat transfer is noted in many studies (see [23,24,25,26,27,28,29] and the references cited therein). The solvability of some models for non-Newtonian fluid flows under slip boundary conditions is proven in [30,31,32,33,34].

Another innovative aspect of our work is that we take into account the viscous dissipation (mechanical-to-thermal energy conversion) in the energy equation. In many papers (for example, see [35,36,37]), the influence of viscous dissipation is neglected because the mathematical analysis of heat and motion equations are considerably simplified due to artificially vanishing the nonlinear Rayleigh function. However, from the physical point of view, it is more interesting not to make this simplification and keep all nonlinearities in the origin equations [38,39,40,41,42].

It should be mentioned at this point that exact solutions for tube and slit flows of a FENE-P fluid with a vanishing solvent viscosity were first given by Oliveira [10] under the no-slip boundary conditions. He considered the situation in which the average velocity (or flow rate) is known, while the pressure gradient is unknown. Later, Cruz et al. [43] obtained analytical solutions corresponding isothermal flows in pipes/channels for two viscoelastic fluids possessing a Newtonian solvent, where the polymer contribution is either described by the Phan-Thien–Tanner or FENE-P models. A unified formulation for velocity profile is presented in [44] for the case of laminar and fully developed flow in both circular and flat ducts. Finally, mention that semi-analytical solutions for the concentric core annular flow of FENE-P-type fluid in horizontal and inclined pipes are found by Guo et al. [45].

## 2. Boundary Value Problems for Poiseuille Flow of FENE-P Fluid

Let us consider the unidirectional steady-state flow of a FENE-P fluid between horizontal plates y=±h due to a constant pressure gradient

(8) $$\frac{\partial p}{\partial x}=-\xi ,\;\xi =\mathrm{const},\;\xi >0,$$

under the assumption that

$$\vec{g}={\left(0,-g,0\right)}^{⊤},\;\theta =\theta \left(y\right),\;\psi \equiv 0,$$

where *g* is the value of acceleration due to gravity. In other words, we deal with the plane Poiseuille flow problem for a FENE-P fluid.

**Figure 1:**
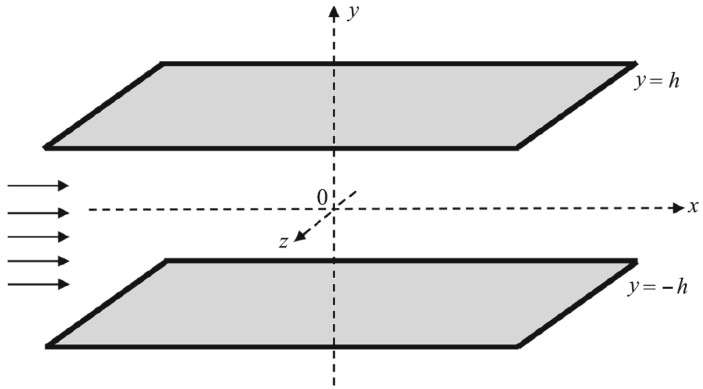
Geometry of the FENE-P fluid flow between the horizontal plates y=±h driven by a constant pressure gradient ∂p/∂x.

The flow configuration and the used Cartesian coordinate system are presented in Figure 1.

In our work, it is assumed that the walls of the channel are unmoved and impermeable.

Also note that the flow model under consideration has the symmetry property with respect to the plane y=0.

### 2.1. Basic Equations

Within the framework of the above assumptions, for the components $v_{x}$, $v_{y}$, $v_{z}$ of the velocity vector $\vec{v}$, we have

(9) $$v_{x}=u\left(y\right),\;v_{y}=0,\;v_{z}=0,$$

where u:[−h,h]→R is an unknown even function.

Moreover, it can easily be checked that the following identities hold:

(10) $$\begin{array}{cc} \frac{\partial \vec{v}}{\partial t}+\mathrm{div}\left(\vec{v}⊗\vec{v}\right) & \equiv \vec{0}, \end{array}$$

(11) $$\begin{array}{cc} \nabla \cdot \vec{v} & \equiv 0, \end{array}$$

(12) $$\begin{array}{cc} \frac{\partial \theta }{\partial t}+\left(\vec{v}\cdot \nabla \right)\theta & \equiv 0, \end{array}$$

(13) $$\begin{array}{cc} \frac{\partial A}{\partial t}+\left(\vec{v}\cdot \nabla \right)A & \equiv 0. \end{array}$$

In view of relations (3), (7), and (10)–(13), general system (1)–(6) reduces to

(14) $$\begin{array}{c} -\nabla p+\mathrm{div}\;S+\rho \vec{g}=\vec{0}, \end{array}$$

(15) $$\begin{array}{c} S=\frac{\eta }{\lambda }\left(\frac{A}{1-\frac{\mathrm{tr}(A)}{L^{2}}}-aI\right), \end{array}$$

(16) $$\begin{array}{c} \left(\nabla \vec{v}\right)A+A{\left(\nabla \vec{v}\right)}^{⊤}=\frac{1}{\lambda }\left(\frac{A}{1-\frac{\mathrm{tr}(A)}{L^{2}}}-aI\right), \end{array}$$

(17) $$\begin{array}{c} -\kappa \theta^{\prime \prime }=\Phi \left(-pI+S,D\left(\vec{v}\right)\right). \end{array}$$

Here and in the sequel, the prime symbol ′ denotes the differentiation with respect to *y*.

### 2.2. Boundary Conditions

We will use a one-parameter Navier-type slip condition on the channel walls y=±h with the parameter *k*. This condition states that the slip velocity is directly proportional to the shear stress in the fluid (see the pioneering work by Navier [46]):

(18) $$\left\{\begin{array}{cc} \vec{v}\cdot \vec{n}=0, & \\ {\left(T\vec{n}\right)}_{\tan}=-k{\vec{v}}_{\tan}, \end{array}\right.$$

where

- $\vec{n}$ is the outer unit normal vector to a plate;
- *k* is the friction coefficient, k≥0;
- ${\vec{v}}_{\tan}$ denotes the component of the vector $\vec{v}$ in the tangential direction at the channel wall, that is, ${\vec{v}}_{\tan}=\vec{v}-\left(\vec{v}\cdot \vec{n}\right)\vec{n}.$

In the limit case when k=0, Navier’s condition passes to the *perfect slip condition* ${\left(T\vec{n}\right)}_{\tan}=\vec{0}$ (see, for example, [47,48,49]), which means that the shear stress at the boundary is zero and the fluid flow behaves like the boundary does not exist. Note that sometimes it is expedient to use the non-zero condition ${\left(T\vec{n}\right)}_{\tan}=\vec{f}$ as a part of mixed boundary conditions for a flow model [50,51,52,53].

On the other hand, if k→+∞, then (18) passes to the standard no-slip condition $\vec{v}=\vec{0}$. Therefore, the Navier slip with k∈(0,+∞) can be considered as an intermediate regime between the perfect slip (k=0) and no-slip (k=+∞) regimes [54]. Carrying this idea further, one can see that (18) is a transformation condition with the homotopy parameter *k*.

We also consider the threshold-type slip condition, assuming that on the channel walls the slipping occurs only when the magnitude of the shear stresses overcomes some threshold value σ:

(19) v→·n→=0,|∥(Tn→)tan∥R3 ≤ σ⟹v→tan=0→,|∥(Tn→)tan∥R3 > σ⟹(Tn→)tan=−σ+k∥v→tan∥R3v→tan∥v→tan∥R3,

where σ is a constant such that σ≥0 and ${∥\cdot ∥}_{R^{3}}$ denotes the Euclidean norm in space $R^{3}$.

In the literature, system (19) is also called the *Navier–Fujita slip condition* [55,56] and the *stick-and-slip boundary condition* [3]. Note that, in the limit case σ=0, this system coincides with Navier’s slip condition (18). If we formally take σ=+∞ in (19), then we arrive at the no-slip regime.

For the temperature function θ, the Robin boundary conditions are prescribed:

(20) $$\kappa \theta^{\prime }\left(\pm h\right)=\mp \beta \theta \left(\pm h\right)$$

with a positive coefficient β characterizing the heat transfer on the channel walls. The above boundary conditions represent Newton’s law of cooling.

## 3. Constructing Exact Solutions

Finding and verification of exact solutions to the above formulated boundary value problems for the flow of a FENE-P fluid are divided into the following seven steps.

**Step 1: Eliminating the configuration tensor A from the governing equations.** We try to eliminate A from relations (14)–(17) and obtain a closed system with respect to the unknowns $\vec{v}$, *p*, S, and θ.

Combining (15) and (16), we find

(21) $$S=\eta \left(\left(\nabla \vec{v}\right)A+A{\left(\nabla \vec{v}\right)}^{⊤}\right).$$

Left-multiplying equality (15) by $\nabla \vec{v}$, we obtain

(22) $$\left(\nabla \vec{v}\right)S=\frac{\eta }{\lambda }\left(\frac{(\nabla \vec{v})A}{1-\frac{\mathrm{tr}(A)}{L^{2}}}-a\nabla \vec{v}\right).$$

Right-multiplying equality (15) by ${\left(\nabla \vec{v}\right)}^{⊤}$ gives

(23) $$S{\left(\nabla \vec{v}\right)}^{⊤}=\frac{\eta }{\lambda }\left(\frac{A{\left(\nabla \vec{v}\right)}^{⊤}}{1-\frac{\mathrm{tr}(A)}{L^{2}}}-a{\left(\nabla \vec{v}\right)}^{⊤}\right).$$

Further, summing equalities (22) and (23), we obtain

$$\left(\nabla \vec{v}\right)S+S{\left(\nabla \vec{v}\right)}^{⊤}=\frac{\eta }{\lambda }\left(\frac{\left(\nabla \vec{v}\right)A+A{\left(\nabla \vec{v}\right)}^{⊤}}{1-\frac{\mathrm{tr}(A)}{L^{2}}}-a(\nabla \vec{v}+{\left(\nabla \vec{v}\right)}^{⊤})\right).$$

Taking into account relation (21), one can derive from the last equality that

$$\left(\nabla \vec{v}\right)S+S{\left(\nabla \vec{v}\right)}^{⊤}=\frac{\eta }{\lambda }\left(\frac{S}{\eta (1-\frac{\mathrm{tr}(A)}{L^{2}})}-a(\nabla \vec{v}+{\left(\nabla \vec{v}\right)}^{⊤})\right).$$

Multiplying both sides of this equality by λ, we arrive at

(24) $$\lambda \left(\left(\nabla \vec{v}\right)S+S{\left(\nabla \vec{v}\right)}^{⊤}\right)=\frac{S}{1-\frac{\mathrm{tr}(A)}{L^{2}}}-a\eta \left(\nabla \vec{v}+{\left(\nabla \vec{v}\right)}^{⊤}\right).$$

Next, applying the trace operator to both sides of equality (15), we obtain

$$\mathrm{tr}\left(S\right)=\frac{\eta }{\lambda }\left(\frac{\mathrm{tr}(A)}{1-\frac{\mathrm{tr}(A)}{L^{2}}}-3a\right).$$

This yields

$$\mathrm{tr}\left(A\right)=\frac{L^{2}\left(\lambda \mathrm{tr}\left(S\right)+3a\eta \right)}{\eta L^{2}+\lambda \mathrm{tr}\left(S\right)+3a\eta },$$

and hence

(25) $$\frac{1}{1-\frac{\mathrm{tr}(A)}{L^{2}}}=\frac{\eta L^{2}+\lambda \mathrm{tr}\left(S\right)+3a\eta }{\eta L^{2}}.$$

Substituting (25) into the right-hand side of equality (24), we obtain

(26) $$\lambda \left(\left(\nabla \vec{v}\right)S+S{\left(\nabla \vec{v}\right)}^{⊤}\right)=\frac{\eta L^{2}+\lambda \mathrm{tr}\left(S\right)+3a\eta }{\eta L^{2}}S-a\eta \left(\nabla \vec{v}+{\left(\nabla \vec{v}\right)}^{⊤}\right).$$

Let us introduce the notation

(27) $$\omega \left(\eta ,\lambda ,a,L,S\right)\overset{\mathrm{def}}{=}\frac{\eta L^{2}+\lambda \mathrm{tr}\left(S\right)+3a\eta }{\eta L^{2}}$$

and rewrite relation (26) as follows:

(28) $$\lambda \left(\left(\nabla \vec{v}\right)S+S{\left(\nabla \vec{v}\right)}^{⊤}\right)=\omega \left(\eta ,\lambda ,a,L,S\right)S-a\eta \left(\nabla \vec{v}+{\left(\nabla \vec{v}\right)}^{⊤}\right).$$

Thus, we have obtained the closed system (14), (17), (28) for finding $\vec{v}$, *p*, S, and θ.

**Step 2: Finding the extra stress tensor** S. Equation (28) is equivalent to the following nonlinear system:

(29) $$\begin{array}{cc} 2\lambda u^{\prime }S_{xy}-\omega \left(\eta ,\lambda ,a,L,S\right)S_{xx} & =0, \end{array}$$

(30) $$\begin{array}{cc} \lambda u^{\prime }S_{yy}-\omega \left(\eta ,\lambda ,a,L,S\right)S_{xy}+a\eta u^{\prime } & =0, \end{array}$$

(31) $$\begin{array}{cc} \lambda u^{\prime }S_{yz}-\omega \left(\eta ,\lambda ,a,L,S\right)S_{xz} & =0, \end{array}$$

(32) $$\begin{array}{cc} \omega \left(\eta ,\lambda ,a,L,S\right)S_{yy} & =0, \end{array}$$

(33) $$\begin{array}{cc} \omega \left(\eta ,\lambda ,a,L,S\right)S_{yz} & =0, \end{array}$$

(34) $$\begin{array}{cc} \omega \left(\eta ,\lambda ,a,L,S\right)S_{zz} & =0. \end{array}$$

We derive from relations (25) and (27) that

$$\omega \left(\eta ,\lambda ,a,L,S\right)=\frac{1}{1-\frac{\mathrm{tr}(A)}{L^{2}}}\neq 0,$$

for any *y* such that −h≤y≤h. Therefore, from (32)–(34), it follows that

$$\begin{array}{cc} S_{yy} & =0,\\ S_{yz} & =0,\\ S_{zz} & =0. \end{array}$$

In addition, if we combine this with (30) and (31), we obtain

(35) $$\begin{array}{c} -\omega \left(\eta ,\lambda ,a,L,S\right)S_{xy}+a\eta u^{\prime }=0,\\ S_{xz}=0. \end{array}$$

Now, we multiply equality (29) by −aη/ω(η,λ,a,L,S) and equality (35) by

$2\lambda S_{xy}/\omega \left(\eta ,\lambda ,a,L,S\right)$. Then we add the results. This gives

(36) $$S_{xx}=\frac{2\lambda }{a\eta }S_{xy}^{2}.$$

It follows from relations (8) and (14) that

$$\frac{\partial S_{xy}}{\partial y}=\frac{\partial p}{\partial x}=-\xi ,$$

whence

$$S_{xy}=-\xi y+c,\;c=\mathrm{const}.$$

Since the flow under consideration is symmetric with respect to the plane y=0, the constant *c* is equal to 0. Therefore, the following equality holds:

(37) $$S_{xy}=-\xi y.$$

Substituting the value of $S_{xy}$ into the right-hand side of equality (36), we obtain

(38) $$S_{xx}=\frac{2\lambda \xi^{2}y^{2}}{a\eta }.$$

Thus, we arrive at the explicit formula for S:

(39) $$S=\left[\begin{array}{ccc} \frac{2\lambda \xi^{2}y^{2}}{a\eta } & -\xi y & 0\\ -\xi y & 0 & 0\\ 0 & 0 & 0 \end{array}\right].$$

**Step 3: Finding the configuration tensor A.** From relation (15) it follows that

$$A=\frac{1}{\omega (\eta ,\lambda ,a,L,S)}\left(\frac{\lambda }{\eta }S+aI\right),$$

whence, taking into account (39) and (27), we obtain

(40) A=|L2(2ξ2λ2y2+a2η2)q(ξ,λ,y,a,L,η)−aηL2λξyq(ξ,λ,y,a,L,η)0|−aηL2λξyq(ξ,λ,y,a,L,η)a2η2L2q(ξ,λ,y,a,L,η)000a2η2L2q(ξ,λ,y,a,L,η)

with

$$q\left(\xi ,\lambda ,y,a,L,\eta \right)\overset{\mathrm{def}}{=}2\xi^{2}\lambda^{2}y^{2}+aL^{2}\eta^{2}+3a^{2}\eta^{2}.$$

By ${\left[A\right]}_{i\times i}$, i=1,2,3, we denote the upper left *i*-by-*i* corner of the matrix A.

The straightforward calculation establishes that

|Tdet([A]1×1)=L2(2ξ2λ2y2+a2η2)q(ξ,λ,y,a,L,η),|TTdet([A]2×2)=a2η2L4(ξ2λ2y2+a2η2)q2(ξ,λ,y,a,L,η),|Tdet([A]3×3)=a4η4L6(ξ2λ2y2+a2η2)q3(ξ,λ,y,a,L,η),

and hence

$$\det({\left[A\right]}_{i\times i})>0,\;\forall \;i=1,2,3.$$

Therefore, applying Sylvester’s criterion, we deduce that the matrix A is positive-definite. This confirms the correctness of the obtained solution (cf. [2,17]).

**Step 4: Finding the velocity vector $\vec{v}$.** Using relations (27), (37), and (38), we derive from equality (35) that

$$u^{\prime }\left(y\right)=-\frac{\xi y(2\lambda^{2}\xi^{2}y^{2}+a\eta^{2}L^{2}+3a^{2}\eta^{2})}{a^{2}\eta^{3}L^{2}}.$$

Integrating the last equality with respect to *y*, we obtain

(41) $$u\left(y\right)=-\frac{\xi y^{2}\left(\xi^{2}\lambda^{2}y^{2}+\left(aL^{2}+3a^{2}\right)\eta^{2}\right)}{2a^{2}L^{2}\eta^{3}}+c,$$

where *c* is a constant.

In accordance with Navier’s slip boundary condition (18), the following equality must be satisfied:

ξh=ku(±h),

or equivalently,

$$\xi h=-\frac{k\xi \left(\xi^{2}\lambda^{2}h^{2}+\left(aL^{2}+3a^{2}\right)\eta^{2}\right)h^{2}}{2a^{2}L^{2}\eta^{3}}+kc,$$

whence

(42) $$c=\frac{\xi h}{k}+\frac{\xi \left(\xi^{2}\lambda^{2}h^{2}+\left(aL^{2}+3a^{2}\right)\eta^{2}\right)h^{2}}{2a^{2}L^{2}\eta^{3}}.$$

Substituting (42) into equality (41), we have

(43) $$u\left(y\right)=\frac{\xi^{3}\lambda^{2}\left(h^{4}-y^{4}\right)+\left(aL^{2}+3a^{2}\right)\xi \eta^{2}\left(h^{2}-y^{2}\right)}{2a^{2}L^{2}\eta^{3}}+\frac{\xi h}{k}.$$

Under the threshold slip boundary condition (19), the velocity component *u* is determined by the following formula:

(44) u(y)=1∫aξ3λ2(h4−y4)+(aL2+3a2)ξη2(h2−y2)2a2L2η3ifξh≤σ,∫a1ξ3λ2(h4−y4)+(aL2+3a2)ξη2(h2−y2)2a2L2η3+ξh−σkifξh>σ.

It is readily seen that combined formula (44) reduces to (43) as $\sigma \to 0^{+}$.

Taking into account (9), we obtain the velocity field:

(45) $$\vec{v}={\left(u(y),0,0\right)}^{⊤},$$

where the function *u* is given by (43) if Navier’s slip regime holds on the channel walls, while formula (44) should be used under the threshold-type slip boundary condition.

**Step 5: Finding the pressure***p*. Using relations (14) and (39), we obtain the explicit formula for the function *p*:

(46) p=−ξx−ρgy+c,c=const.

**Step 6: Finding the temperature θ.** In order to find the temperature distribution θ, we must solve Equation (17) under boundary condition (20).

First, let us calculate the value of Φ. Taking into account (9) and (39), we obtain

$$D\left(\vec{v}\right)=\left[\begin{array}{ccc} 0 & \frac{1}{2}u^{\prime } & 0\\ \frac{1}{2}u^{\prime } & 0 & 0\\ 0 & 0 & 0 \end{array}\right],$$

$$-pI+S=\left[\begin{array}{ccc} \frac{2\lambda \xi^{2}y^{2}}{a\eta }-p & -\xi y & 0\\ -\xi y & -p & 0\\ 0 & 0 & -p \end{array}\right],$$

and hence

(47) $$\begin{array}{cc} \Phi (-pI+S,D\left(\vec{v}\right)) & =\frac{1}{c_{p}}\left(-pI+S\right):D\left(\vec{v}\right)\\ \; & =-\frac{\xi yu^{\prime }}{c_{p}}\\ \; & =\frac{\xi^{2}\left(2\lambda^{2}\xi^{2}y^{2}+a\eta^{2}L^{2}+3a^{2}\eta^{2}\right)y^{2}}{a^{2}\eta^{3}L^{2}c_{p}}. \end{array}$$

Substituting (47) into (17), we arrive at the following equation

$$-\kappa \theta^{\prime \prime }\left(y\right)=\frac{\xi^{2}\left(2\lambda^{2}\xi^{2}y^{2}+a\eta^{2}L^{2}+3a^{2}\eta^{2}\right)y^{2}}{a^{2}\eta^{3}L^{2}c_{p}}.$$

Solving this equation subject to boundary condition (20) with respect to the function θ, we obtain

(48) |Aθy=−4λ2ξ4y6+5ξ2η2aL2+3a2y460κcpa2η3L2|AA+ξ2h44h2ξ2λ2+5L2aη2+15a2η260κcpa2η3L2|A+ξ2h315a2η2+6h2ξ2λ2+5L2aη215βcpa2η3L2.

**Step 7: Verification of the obtained solutions.** The direct substitution of relations (39), (40), (45), (46), and (48) into system (14)–(17) with boundary conditions (18) and (20) (respectively, (19) and (20)) shows that $\vec{v}$, *p*, S, A, and θ satisfy this system.

## 4. Discussion

As can be seen from the previous section, we have obtained explicit formulas for the calculation of the velocity field, the pressure, the extra stresses, the configuration tensor, and the temperature distribution. Thus, the problem under consideration is completely solved in the analytical form for both Navier’s slip and threshold-type slip boundary conditions.

Since, in the limit case k→+∞, boundary condition (18) reduces to the no-slip condition $\vec{v}=\vec{0}$, our analysis is also suitable for this boundary regime. Indeed, it can easily be checked that the velocity field ${\vec{v}}_{0}={\left(u_{0}\left(y\right),0,0\right)}^{⊤}$ with

(49) $$u_{0}\left(y\right)\overset{\mathrm{def}}{=}\frac{\xi^{3}\lambda^{2}\left(h^{4}-y^{4}\right)+\left(aL^{2}+3a^{2}\right)\xi \eta^{2}\left(h^{2}-y^{2}\right)}{2a^{2}L^{2}\eta^{3}}$$

corresponds to the Poiseuille flow of the FENE-P fluid that obeys the no-slip condition on the channel walls. Note that, in contrast to a Newtonian fluid taking a parabolic velocity profile in such flows, the velocity distribution $u_{0}$ is determined by a fourth-degree polynomial. This is consistent with the results by Oliveira [10].

Using (49), one can rewrite formula (44) in the compact form:

(50) u(y)=u0(y)ifξh≤σ,∫a1u0(y)+ξh−σkifξh>σ.

Clearly, the product ξh is one of the key parameters for the model under consideration. If ξh overcomes the threshold value σ, then the slip regime arises at solid surfaces; otherwise, the fluid adheres to the channel walls.

Since the following equality

$$a=\frac{1}{1-\frac{3}{L^{2}}}$$

holds, we have

(51) $$a\to 1^{+}\;asL\to +\infty .$$

Therefore, in the case when L→+∞ (infinite extensibility), we derive from (50) the velocity solution

(52) uM(y)=ξ2η(h2−y2)ifξh≤σ,∫a1ξ2η(h2−y2)+ξh−σkifξh>σ,

which is parabolic as for a Newtonian fluid. The subscript *M* in the left-hand side of (52) means that we are dealing with a Maxwell-type viscoelastic medium [57,58,59]. Indeed, we claim that, in this limit case, the used rheological relations reduce to the constitutive equation of the upper convected Maxwell model.

Note that if L→+∞, then (4) and (5) pass to

(53) $$S=\frac{\eta }{\lambda }\left(A-I\right)$$

and

$$\overset{\nabla }{A}=-\frac{1}{\lambda }\left(A-I\right),$$

respectively. Moreover, combining the above equations, one can derive

(54) $$S=-\eta \overset{\nabla }{A}.$$

Applying the operator of Oldroyd’s upper convected derivative to both parts of relation (53), we obtain

(55) $$\begin{array}{cc} \overset{\nabla }{S} & =\frac{\eta }{\lambda }\left(\overset{\nabla }{A}-\overset{\nabla }{I}\right)\\ \; & =\frac{\eta }{\lambda }\left(\overset{\nabla }{A}+2D\left(\vec{v}\right)\right)\\ \; & =\frac{\eta }{\lambda }\overset{\nabla }{A}+\frac{2\eta }{\lambda }D\left(\vec{v}\right), \end{array}$$

where we used the equality

$$\overset{\nabla }{I}=-2D\left(\vec{v}\right),$$

which holds in the situation of the flow with the only non-zero velocity component $v_{x}=u\left(y\right)$.

From (55) it follows that

$$\lambda \overset{\nabla }{S}=\eta \overset{\nabla }{A}+2\eta D\left(\vec{v}\right).$$

In view of formula (54), the last relation can be rewritten as follows:

$$\lambda \overset{\nabla }{S}+S=2\eta D\left(\vec{v}\right),$$

that is, we have arrived at the constitutive equation of a Maxwell fluid.

**Figure 2:**
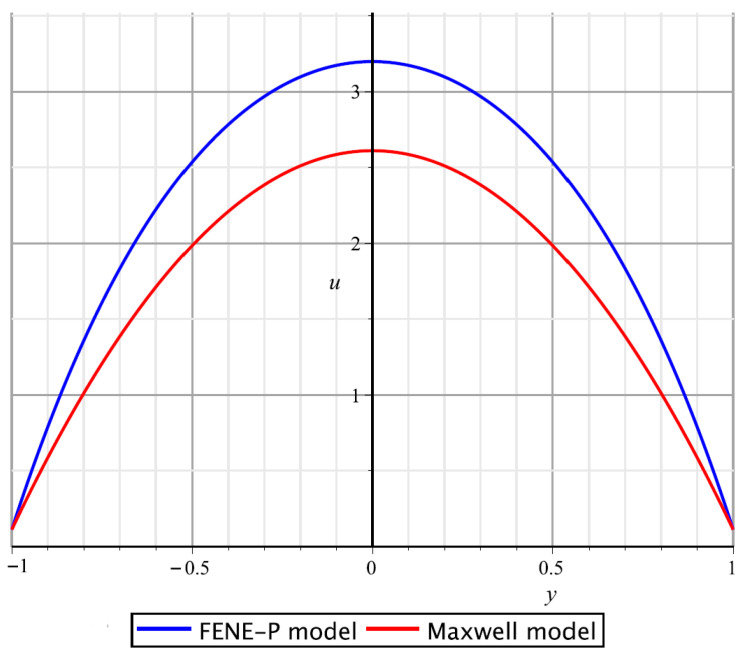
Velocity profiles for the Poiseuille flow under the assumption that h=1, L=10, ξ=5, λ=1, η=1, σ=4, and k=9.

An example of velocity profiles in the Poiseuille flow for both the FENE-P and Maxwell models, which are obtained by the constructed exact solutions (see formulas (50) and (52)), is given in Figure 2.

Now, we pass to the limit L→+∞ in formula (48) for the temperature θ. Taking into account (51), we obtain

(56) $$\theta_{M}\left(y\right)=\frac{\xi^{2}}{12\kappa c_{p}\eta }\left(h^{4}-y^{4}\right)+\frac{\xi^{2}h^{3}}{3\beta c_{p}\eta }.$$

This function describes the temperature distribution in the flow of the Maxwell fluid with the corresponding material constants.

**Figure 3:**
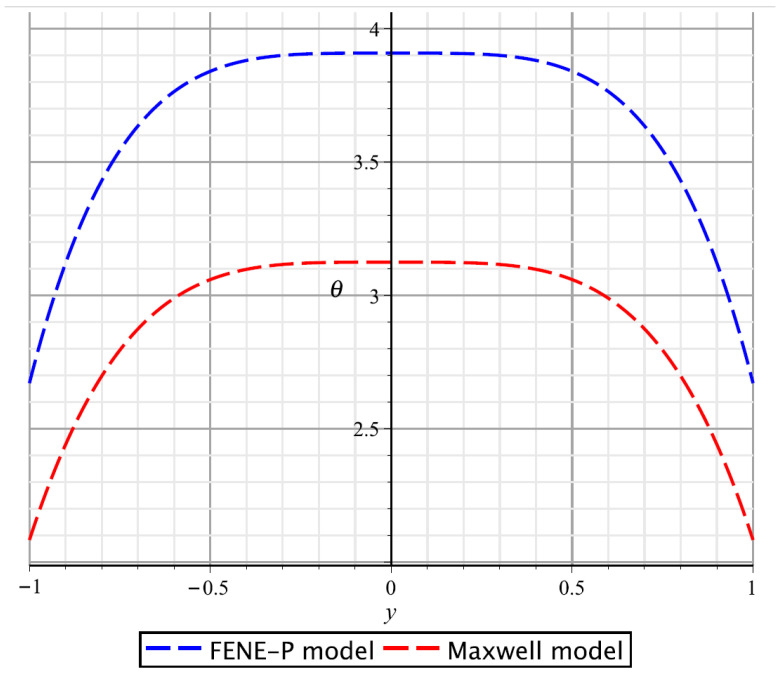
Temperature profiles for the Poiseuille flow under the same parameters as on Figure 2 and κ=2, β=4, $c_{p}=1$.

If we introduce the simplifying assumption Φ≡0 and insert it into the heat transfer Equation (6), then instead of formulas (48) and (56), we would obtain the zero temperature distribution. Figure 3 clearly demonstrates that this assumption on the vanishing Rayleigh function is not always justified since, in fact, the temperature profile may differ significantly from zero. Therefore, when modeling flows of heat-conducting viscoelastic fluids, it is advisable to take into account the viscous dissipation effect, that is, the conversion of mechanical energy to thermal energy.

## 5. Conclusions

In this article, we have investigated the boundary value problems describing the steady-state, non-isothermal, pressure-driven flow of an incompressible viscoelastic fluid of a FENE-P-type in the horizontal channel with impermeable solid walls. The viscous dissipation effect as well as Navier’s and threshold-type velocity slip at the channel walls are included in our analysis. The full analytical solution is derived in the framework of elementary functions. Namely, new explicit formulas for the calculation of the velocity field, the temperature distribution, the pressure, the extra stresses, and the configuration tensor of a FENE-P fluid are obtained. The case when the extensibility parameter tends to infinity is discussed separately. Also, explicit relationships are for the model parameters that determine which regime is realized at the boundary of the flow domain: no-slip or slip regime.

An interesting direction for further research of FENE-P fluids is to develop the results of this paper to other kinds of flows, for example, to the case of helical flows [60,61] as well as to time-dependent motions. The known and new classes of exact solutions can be used to computational fluid dynamics. They can also serve as the basis for improving the packages of applied software for engineering and scientific calculations in models described by partial differential equations.

## Data Availability

Not applicable.
